# Real-world treatment patterns and outcomes in severe acute exacerbations of chronic obstructive pulmonary disease: an observational study

**DOI:** 10.1186/s12890-026-04599-z

**Published:** 2026-08-14

**Authors:** Lilla Árvai-Horváth, Piroska Manzel, István Méhész, Dalma Wessják, Dávid Á. Karádi, Szabolcs Baglyas, András Lorx, Luca Valkó

**Affiliations:** 1 Doctoral College, Semmelweis University, Üllői Út 26. Fsz. 9, Budapest, 1085 Hungary; 2 Department of Anaesthesiology and Intensive Therapy, Bács-Kiskun County Teaching Hospital, Nyíri Út 38, Kecskemét, 6000 Hungary; 3 Department of Anaesthesiology and Intensive Therapy, Borsod-Abaúj-Zemplén County Central Hospital and University Teaching Hospital, Szentpéteri kapu 72–76, Miskolc, 3526 Hungary; 4 Department of Anaesthesiology and Intensive Therapy, Uzsoki Street Hospital, Róna u 196-212, Budapest, 1146 Hungary; 5 Department of Anaesthesiology and Intensive Therapy, Bajcsy-Zsilinszky Hospital and Outpatient Clinic, Maglódi út 89–91, Budapest, 1106 Hungary; 6 Department of Anaesthesiology and Intensive Therapy, Semmelweis University, Üllői út 78/B, Budapest, H-1082 Hungary

**Keywords:** COPD, AECOPD, Ventilatory failure, Noninvasive ventilation, NIV failure, Hospital mortality

## Abstract

**Background:**

Chronic obstructive pulmonary disease (COPD) represents a significant healthcare burden. Acute exacerbation (AECOPD) episodes frequently require hospitalization, and a number of these involve intensive care (ICU) unit admission. Noninvasive ventilation (NIV) use is associated with superior outcome, despite this, real-world data indicate that NIV remains underutilized. Our aim was to characterize NIV utilization for severe AECOPD patients admitted to participating Hungarian ICUs.

**Methods:**

We conducted a 2-month long prospective observational study of AECOPD patients in ICUs and collected national health registry data for parallel validation.

**Results:**

In five participating ICUs 27 (4.86%) out of 555 screened patients were recruited. Patients were predominantly older, overweight, and had several comorbidities. Hypercapnia, elevated bicarbonate levels and pneumonia were common at admission. ICU mortality was 22.2%, and in-hospital mortality rate reached 37%, compared to a predicted 55 (25–73%) ICU mortality.

NIV was used in most patients, however, only 25.9% received NIV exclusively. NIV was the initial ventilation type in 55.6% of cases, but early NIV failure rate was 46.7%. Difficult weaning was reported in 30% of patients. NIV failure was associated with longer ICU stay and increased hospital mortality.

National Health Fund data confirmed overall low NIV use, with only 4.8% of COPD patients receiving this therapy.

**Conclusions:**

Severe AECOPD in Hungarian ICUs is currently associated with low NIV utilization, high NIV failure rate and considerable hospital mortality. Future studies should focus on appropriate patient selection, initial NIV application and therapy optimization.

**Supplementary Information:**

The online version contains supplementary material available at https://doi.org/10.1186/s12890-026-04599-z.

## Background

Chronic obstructive pulmonary disease (COPD) represents a significant healthcare burden, with 30–50% of affected patients experiencing at least one acute exacerbation (AECOPD) per year [1–4]. Approximately 20% of AECOPD episodes require hospitalization, and up to 19% of these involve intensive care unit (ICU) admission, corresponding to over three million cases annually worldwide [3, 5, 6].

Studies have shown that noninvasive ventilation (NIV) as first-line therapy is advantageous, particularly as the initial treatment of patients with pH below 7.35, but also as a step-down strategy during weaning from invasive mechanical ventilation (IMV) [7]. Despite this, real-world data indicate that NIV therapy efficacy and failure rates vary considerably across centers, and overall, NIV remains underutilized, although its use may be increasing in some regions [1, 8–14].

There is a lack of standardized clinical guidelines specifically addressing NIV initiation, optimization of ventilator settings, or determining well-defined criteria for escalation to IMV. Moreover, despite the presence of dedicated NIV units, ICUs continue to serve as the primary treatment setting for most severe cases of AECOPD [15]. Hospital mortality of these most severe cases ranges between 17–49% and current understanding of factors influencing patient outcomes following ICU admission due to severe AECOPD remains limited [3].

The primary objective of the present study was to characterize NIV utilization and failure rate in severe AECOPD patients admitted to participating Hungarian ICUs. Secondary outcomes included assessment of weaning outcomes, ICU and hospital mortality, and factors associated with adverse outcomes.

## Methods

### Study design and setting

We conducted a prospective, observational cohort study involving patients admitted to ICUs with severe AECOPD as their primary diagnosis for treatment. We contacted a total of 10 ICUs with diverse caseloads and high patient volumes to offer voluntary participation in the study. No standardized clinical protocols were implemented within the study framework. Decisions regarding NIV initiation, ventilator settings, escalation to IMV, and weaning were made according to local practice and the treating physician's judgement. Data collection was performed by a designated study physician at each site using a secure, password-protected online questionnaire. Data were recorded pseudonymized to ensure privacy. Data were collected from February 1, 2025. to March 31, 2025.

### Patients and data collected

Inclusion criteria for the study were patients above the age of 18 and admission to a participating ICU with respiratory failure due to severe AECOPD. All ICU admissions were screened for eligibility. COPD was defined as either a previously documented diagnosis or a probable diagnosis of COPD based on a history of tobacco use, chronic sputum production and elevated arterial bicarbonate levels [16, 17]. Severe AECOPD was defined as documented admission diagnosis of COPD and respiratory failure. Exclusion criteria included lack of diagnosed or suspected COPD as the cause for ICU admission and refusal to participate in the study.

We collected the following data: demographics (age, sex, body mass index [BMI]), comorbidities, vital parameters, laboratory values, arterial blood gas values, and Acute Physiology and Chronic Health Evaluation II (APACHE II) score at admission, characteristics of mechanical ventilation (mode and duration of ventilation, need for conversion, need for reintubation). Level of consciousness at admission was assessed using the Glasgow Coma Scale (GCS), a previously published tool for evaluating consciousness based on eye opening, verbal response, and motor response [18]. Sex was recorded as documented and was analyzed descriptively. Primary outcomes included NIV and IMV utilization, length, and NIV failure rate (defined as need for conversion from NIV to IMV). Early NIV failure was defined as conversion need within 48 h of NIV initiation, late NIV failure was defined as after 48 h of NIV initiation [19, 20] Secondary outcomes included mortality (ICU and in-hospital), ICU length of stay, difficult weaning (defined as reintubation within 48 h after extubation, or two or more failed extubation attempts), and extracorporeal CO_2_ utilization) [20, 21].

### External data comparison

To contextualize our findings, we obtained data from the National Health Insurance Fund (NHIF) to provide a healthcare system-level comparison regarding ventilatory support utilization in Hungary in the same period as our observational study. We requested nationwide figures on COPD-related hospital admission (defined as International Classification of Diseases-10 code J4400, J4410, J4480, J4490, and J9600, J9690) during the study period, including mortality rates, presence of acute respiratory failure as a co-diagnosis, and use of NIV or IMV (defined as Current Procedural Terminology code 87,125, 87,126 or 87,120) as documented therapies.

### Statistical analysis

Data are presented as median (25-75th percentile) for continuous variables and N(%) for categorial variables. The Shapiro–Wilk test was applied for normality testing. The Mann–Whitney U test was used to compare continuous variables, and the Fisher exact test for categorical variables. Correlations were tested using the Spearman rank correlation. No a priori sample size calculation was performed because this was an exploratory pilot study conducted during a predefined two-month enrollment period. Missing data were not imputed in the analysis. Due to the limited sample size, no sensitivity analyses was performed. A p-value < 0.05 was considered statistically significant. Statistical analysis was performed using SPSS Version 30.0.0.0 (IBM Corp., Armonk, NY, USA).

## Results

### Study population

**Fig. 1:**
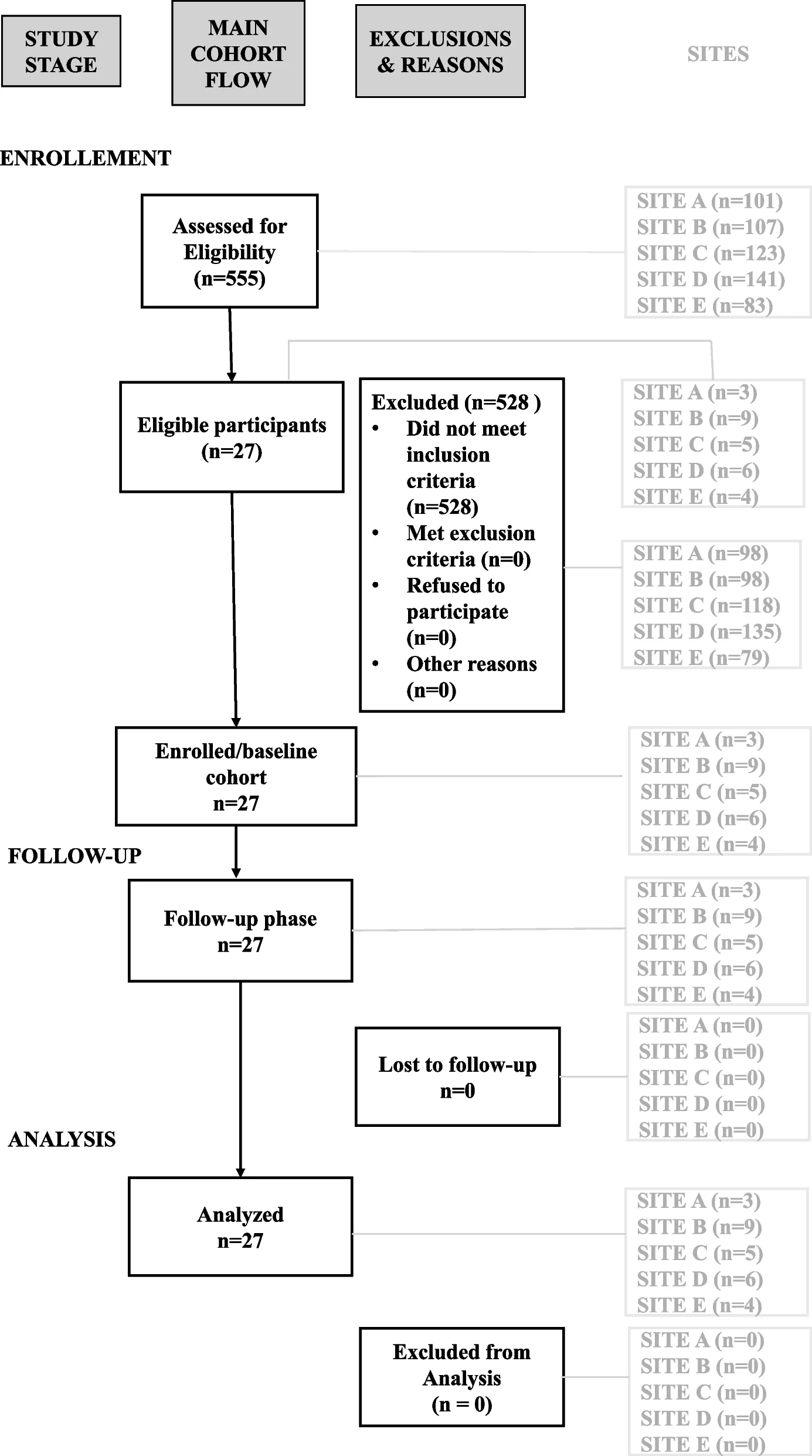
Flowchart of patient enrollment. Flowchart showing the number of study sites, screened patients, excluded patients and enrolled patients, with site-specific patient numbers

Five units provided data for our study: two affiliated with regional hospitals, two with city hospitals, and one with a teaching hospital. Out of 555 patients screened, 27 patients (4.86%) were included in the study (see Fig. 1). Out of the 27 patients, 23 had previously established diagnosis of COPD, while 4 were probable COPD based on admission data and history.

Patient characteristics and baseline clinical data are shown in Table 1. Patients were predominantly over the age of 65, overweight and evenly distributed by sex. Comorbidities were frequent, 18.5%, 48.1%, 48.1% and 25.9% of participants also had myocardial infarction, chronic heart failure, peripheral vascular disease or diabetes mellitus, respectively.

**Table 1:** Patient characteristics and baseline data for all patients and for in-hospital survivor and non-survivor subgroups

	All patients *N =* 27	Survivors *N =* 17	Non-survivors *N =* 10	*p*-value
Age (years)	68 (62–74)	66 (60–71)	72.5 (70–80)	**0.046**
Sex:				
Female	13 (48.1%)	8 (47.1%)	5 (50%)	0.883
Male	14 (51.9%)	9 (52.9%)	5 (50%)	
BMI (kg/m^2^)	29.4 (24.2–36.7)	29.32 (24.2–37.6)	29.39 (25.3–31.9)	0.639
Mean arterial pressure (mmHg)	74.3 (62.0–91.0)	74.33 (63.00–91.00)	74 (50.7–91)	0.570
Heart rate (/min)	97 (70–109)	98 (80–110)	81 (60–104)	0.155
pH	7.29 (7.19–7.3)	7.289 (7.19–7.30)	7.28 (7.19–7.29)	0.902
PaO_2_ (mmHg)	68.7 (60.4–88.4)	75.8 (67.0–127.0)	66 (57.2–75.8)	0.103
PaCO_2_ (mmHg)	76.5 (52.3–99.9)	80 (58–109)	67.75 (45.7–91.7)	0.204
HCO_3_^−^ (mmol/L)	32.5 (27.5–37.9)	32.4 (27.5–39.0)	31.4 (28.6–34.3)	0.386
Respiratory rate (/min)	23 (18–30)	26 (20–30)	19 (17–23)	0.155
FiO_2_ (%)	60 (40–95)	60 (40–75)	87.5 (50–100)	0.115
GCS	13 (3–15)	14 (12–15)	3 (3–11)	**0.027**
Presence of comorbidities:				
Yes	22 (84.6%)	13 (81.3%)	9 (90%)	0.547
No	4 (15.4%)	3 (18.8%)	1 (10%)	
Presence of pneumonia at admission:				
Yes	13 (48.1%)	8 (47.1%)	5 (50%)	0.883
No	14 (51.9%)	9 (52.9%)	5 (50%)	
APACHE II Score	26 (18–30)	22 (17–27)	29 (21–33)	**0.040**

Values are presented as median [interquartile range] or number (%), as appropriate. Comorbidity data were missing for one patient

*BMI* Body mass index, *PaO₂* arterial partial pressure of oxygen, *PaCO₂* arterial partial pressure of carbon dioxide, *HCO₃⁻* bicarbonate, *FiO₂* Fraction of inspired oxygen, *GCS* Glasgow Coma Scale, *APACHE II* Acute Physiology and Chronic Health Evaluation II, *N* Number

Bold values indicate statistical significance (*p* < 0.05)

Initial blood gas parameters were consistent with severe AECOPD, with most patients exhibiting marked acidosis, hypercapnia and elevated bicarbonate levels. Oxygen levels were more varied, although most patients received supplemental oxygen at the time of admission. Cardiovascular involvement was common. Alteration of consciousness was also frequent, although GCS scores indicated only mild impairment in most patients. Pneumonia was present in approximately half of patients at admission. Overall, APACHE II scores reflected a predicted nonoperative mortality of 55 (25–73)%, consistent with a critically ill cohort.

### Primary outcomes

Regarding the primary outcome, NIV was used in the majority of patients during the course of their treatment, however, only a quarter of patients received this mode of ventilation exclusively. Exclusive NIV treatment was observed only among survivors. The initial ventilation type was NIV in over half of cases, however more than half of these resulted in NIV failure. Most conversions from NIV to IMV took place within the first 48 h of starting initial treatment. No NIV failures were observed when NIV was used after IMV. Median NIV duration accounted for 25% of the total length of ventilation for the cohort. Difficult weaning was reported in nearly one third of patients. Detailed treatment data are shown in Table 2.

**Table 2:** Treatment and outcome data for all patients and for in-hospital survivor and non-survivor subgroups

	All patients ***N =* 27**	Survivors ***N =* 17**	Non-survivors ***N =* 10**	*p*-value
Mechanical ventilation used	27 (100%)	17 (100%)	10 (100%)	**0.045**
NIV	7 (25.9%)	7 (41.2%)	0 (0%)	
IMV	5 (18.5%)	2 (11.8%)	3 (30%)	
Combined NIV + IMV	15 (55.6%)	8 (47.1%)	7 (70%)	
Duration of NIV (days)	1 (1–2)	2 (1–2)	1 (0–1)	**0.046**
Duration of IMV	3 (0–11)	2 (0–5)	4.5 (2–13)	0.187
Total length of mechanical ventilation	6 (2–12)	2 (6–7)	7 (2–14)	0.604
Proportion of NIV to total length of ventilation	25 (3.7–100)	66.7 (11.5–100)	7.1 (0–31.3)	**0.018**
ICU length of stay	6 (4–11)	5 (4–7)	9.5 (4–13)	0.639
Initial mode of ventilation:				
NIV	15 (55.6%)	10 (58.8%)	4 (40%)	0.345
IMV	12 (44.4%)	7 (41.2%)	6 (60%)	
Need for conversion any time:	***N =* 22**	***N =* 15**	***N =* 7**	
	8 (36.4%)	4 (26.7%)	4 (57.1%)	0.343
Early conversion (first 48 h):	***N =* 15**	***N =* 11**	***N =* 4**	
	7 (46.7%)	3 (27.3%)	4 (100%)	**0.026**
Late conversion (> 48 h)	***N =* 15**	***N =* 11**	***N =* 4**	
	1 (6.7%)	1 (9.1%)	0 (0%)	1.000
Difficult weaning:	***N =* 20**	***N =* 10**	***N =* 10**	
	6 (30%)	3 (30%)	3 (30%)	1.000
Extracorporeal CO_2_ removal (ECCO_2_R) treatment:	***N =* 27**	***N =* 17**	***N =* 10**	
	0 (0%)	0 (0%)	0 (0%)	1.000

Values are presented as median [interquartile range] or number (%), as appropriate

*NIV* Noninvasive ventilation, *IMV* Invasive mechanical ventilation, *N* Number

Bold values indicate statistical significance (*p*< 0.05)

### Secondary outcomes

The median ICU length of stay was 6 days (4–11). ICU mortality was 22.2% (6/27, 95% CI, 8.6–42.3%) while cumulative in-hospital mortality rate reached 37% (10/27, 95% CI 19.4–57.6%). The observed hospital mortality rate corresponded with the median predicted mortality rate based on the APACHE II score. When comparing outcome groups in our study, non-survivors were older and had lower GCS and higher APACHE II scores than survivors (Table 1). Length of NIV was shorter in the non-survivor group. The ratio of NIV to total length of ventilation was significantly higher in survivors. Among patients initially treated with NIV, early conversion in the first 48 h was more frequent in the non-survivor group. There was no significant difference in initial ventilation mode,or occurrence of difficult weaning between the two groups (Table 2).

**Fig. 2:**
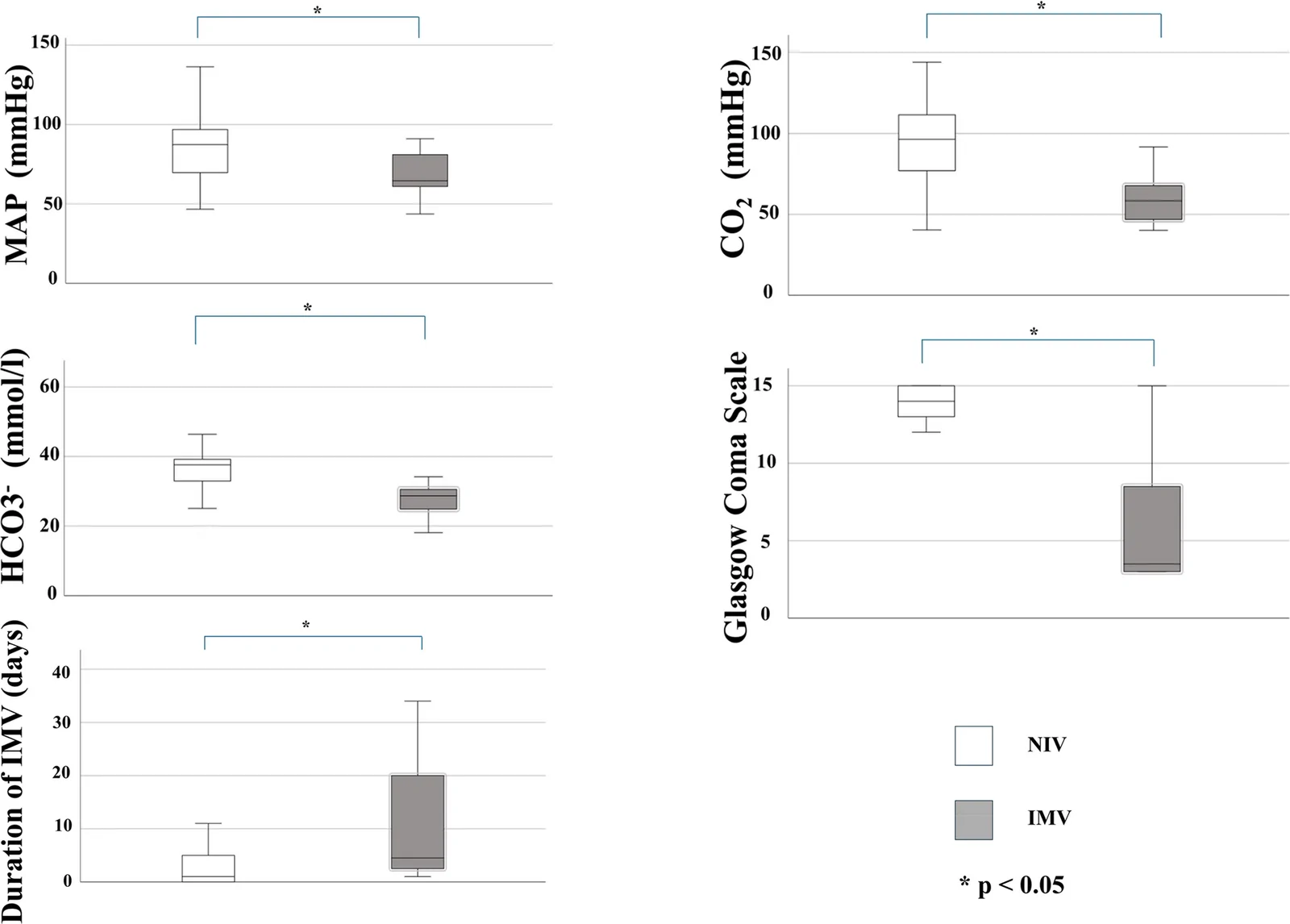
Comparison of initial ventilation modality (boxplot of relevant parameters). Comparison of baseline parameters of patients initially treated with NIV or IMV. Duration of IMV is reported only for patients who received IMV. Boxes represent the interquartile range (25–75.^th^ percentile), the horizontal lines represent the median, whiskers represent the minimum and maximum values. *p <* 0.05 was considered statistically significant. (NIV: noninvasive ventilation, IMV: invasive mechanical ventilation, HCO₃⁻: bicarbonate, CO₂: arterial partial pressure of carbon dioxide, MAP: Mean arterial pressure)

When comparing patients based on the mode of initial ventilation, those who received IMV as a starting treatment had lower mean arterial blood pressure [62.3 (55–69.3) vs 91 (74.3–102) mmHg, *p =* 0.047], lower PaCO_2_ levels (58.7 (51.9–71.6) vs 89.25 (76.5–109) mmHg, *p =* 0.05], lower HCO_3_^−^ levels (29.3 (27.5–33.5) vs 36.9 (29.3–39) mmol/l, *p =* 0.002] and lower GCS scores [3 (3–6) vs 14 (13–15), *p =* 0.04], and received IMV for a longer duration (5 (3–14) vs 0.5 (0–5), *p =* 0.037). (See Fig. 2 and Supplementary Table 1).

When examining factors influencing NIV failure, we found that NIV failure only occurred in cases where NIV was used as the initial ventilation mode, but never if used as step-down therapy after IMV. NIV failure patients had higher heart rate, but no other significant differences in clinical parameters or baseline characteristics, however conversion need at any time was associated with longer ICU stay and a tendency for increased hospital mortality (see Table 3).

**Table 3:** Impact of NIV failure in patients initially treated with NIV

	**No need for conversion** *N =* 7	**Need for conversion anytime** *N =* 8	*p*-value
Age (years)	67.0 (55.0–74.0)	68.5 (58.5–72.5)	1.000
Sex:			
Female	3 (42.9%)	4 (50%)	1.000
Male	4 (57.1%)	4 (50%)	
BMI (kg/m^2^)	34.0 (25.2–39.0)	26.3 (22.9–36.1)	0.281
Mean arterial pressure (mmHg)	91.0 (73.3–102.0)	85.2 (51.8–105.9)	0.536
Heart rate (/min)	70.0 (66.0–93.0)	104.5 (96.2–122.3)	**0.014**
PaO_2_ (mmHg)	68.1 (59.8–88.4)	65.6 (54.1–192.0)	0.867
PaCO_2_ (mmHg)	82.1 (53.6–114.0)	98.2 (78.1–114.3)	0.694
pH	7.3 (7.2–7.3)	7.2 (7.2–7.3)	0.955
Respiratory rate (/min)	29.0 (15.0–32.0)	23.0 (15.0–31.5)	0.779
FiO_2_ (%)	50.0 (40.0–60.0)	72.5 (42.5–95.0)	0.189
HCO_3_^−^ (mmol/L)	32.4 (25.1–40.0)	37.8 (34.8–39.0)	0.536
GCS (3–15)	14.0 (13.0–15.0)	13.5 (5.3–15.0)	0.397
APACHE II Points	17.0 (14.0–29.0)	21.5 (18.8–30.5)	0.281
Duration of NIV (days)	2.0 (1.0–2.0)	1.0 (1.0–1.8)	0.336
ICU length of stay	4.0 (2.0–5.0)	7.5 (5.3–12.5)	**0.040**
Difficult weaning:			
Yes	0 (0%)	2 (25%)	0.467
No	7 (100%)	6 (75%)	
ICU survival:			
Survivor	7 (100%)	5 (62.5%)	0.200
Non-survivor	0 (0%)	3 (37.5%)	
In-hospital survival:			
Survivor	7 (100%)	4 (50%)	0.077
Non-survivor	0 (0%)	4 (50%)	

Values are presented as median [interquartile range] or number (%), as appropriate

*BMI* Body Mass Index, *HCO₃⁻* bicarbonate, *PaO₂* arterial partial pressure of oxygen, *PaCO₂* arterial partial pressure of carbon dioxide, *FiO₂* Fraction of inspired oxygen, *GCS* Glasgow Coma Scale, *APACHE II* Acute Physiology and Chronic Health Evaluation II, *ICU* Intensive care unit, *NIV* Noninvasive ventilation, *N* Number

Bold values indicate statistical significance (*p*< 0.05)

In patients requiring conversion we found no correlation between baseline values and timing of the conversion (data not shown).

### National Health Insurance Fund data review

The NHIF query uncovered a total of 3281 patients admitted to any in-patient service with COPD as the main diagnosis nationwide during the study period. Overall mortality was 12.4% (408 patients). Out of all patients admitted with COPD only 9.2% (301) patients received mechanical ventilation. Out of the 301 patients requiring ventilation, 44.2% (133 patients) had NIV, 47.2% (142 patients) had IMV, and 8.6% (26 patients) received both. Interestingly, mortality rate for patients requiring ventilation was 13.6% (41 patients out of 301), 6.8% (9 out of 133) for patients receiving NIV, 21.1% (30 out of 142) for patients receiving IMV and 7.6% (2 out of 26) for patients receiving both.

For patients with acute respiratory failure as a co-diagnosis (6.9%, 227 out of 3281 patients), only 31 (13.7%) received mechanical ventilation (29.0%—9 out of 31—NIV, 71.0%—22 out of 31—IMV, and 6.5%—2 out of 31—both), but none of them were reported as deceased. The reported mortality rate for the whole group was 2.2% (5 out of 227 patients).

**Fig. 3:**
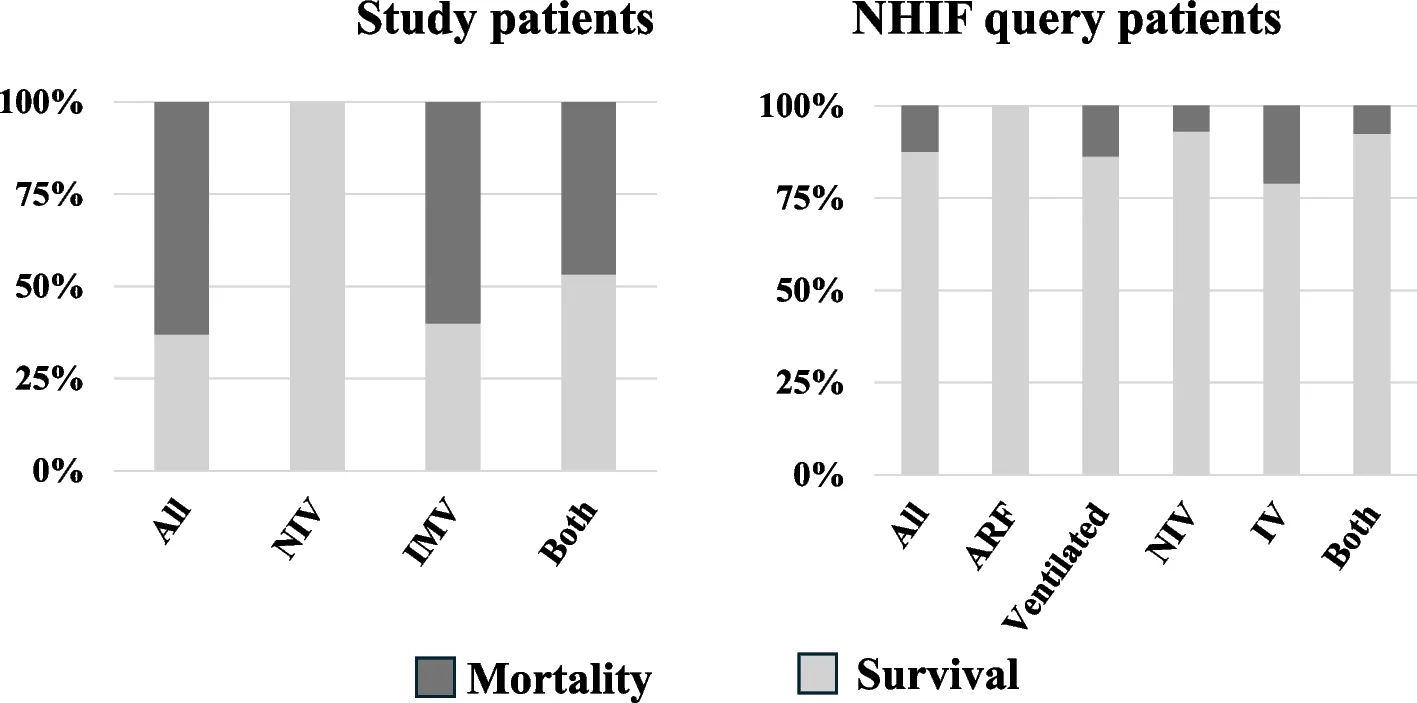
Outcome data of study patients and NHIF query patients. Outcome of different patient groups based on study data and National Health Insurance Fund inquiry data. (NHIF: National Health Insurance Fund, NIV: Noninvasive ventilation, IMV: Invasive mechanical ventilation, ARF: Acute respiratory failure)

Outcome of study and NHIF query patients are demonstrated on Fig. 3.

## Discussion

This prospective, observational, multicenter cohort study evaluated NIV utilization, NIV failure and clinical outcomes in patients with severe AECOPD admitted to Hungarian ICUs. The primary findings were that NIV was widely available within participating ICUs, being used in more than 80% of patients at some point during treatment, but NIV failure occurred frequently, with nearly half of initially treated patients requiring conversion to IMV. Although ICU mortality was 22.2%, in-hospital mortality was considerably higher at 37%, highlighting the healthcare burden of severe AECOPD. Furthermore, nationwide claims data comparison for the study period revealed low overall NIV utilization, with less than 5% of eligible patients receiving this therapy, highlighting potential gaps in care for severe AECOPD.

The relatively low occurrence of AECOPD cases in our study is surprising compared to the worldwide prevalence of COPD, which was estimated to be 10.3% in 2019; and compared to recent large Austrian study that found 8.6% of ICU patients were affected by the disease [1–3, 22]. This discrepancy could be caused by the seasonal fluctuation of exacerbations and the relatively short study period in our study, but also the recent establishment of dedicated NIV units in the country [23]. Furthermore, clinician bias and prognostic pessimism associated with this chronic condition may also contribute [24]. Indeed, the NHIF comparison part of our study reflected larger case numbers, but less than 10% of patients received documented ventilation therapy. Based on these results, the ICU cases do not merely reflect NIV failure and borderline NIV indication cases, while other cases are adequately managed on NIV in other wards. This lack of advanced therapy utilization is in contradiction to data showing that patients receiving mechanical ventilation due to AECOPD show a significantly lower mortality rate compared with those ventilated for other causes [25]. A recent study analyzing data from 302 patients reported an ICU-mortality rate of 23% and one-year-mortality rate of 41% in this patient group [26]. In our cohort ICU mortality was similarly favorable (22.2%), but in-hospital mortality was relatively high. This is in line with a Hungarian study of more than 60,000 patients that found poor long-term survival of COPD patients that were hospitalized with severe acute exacerbations [27]. Other potential explanations for high in-hospital mortality could include greater case severity, high co-occurrence of pneumonia as well as high NIV failure rate. Our findings underscore the need for step-down units and individualized care for these patients.

The advancements made in AECOPD management and improvements in outcomes are largely due to the vast evidence on the use of NIV in this patient population, which may reduce mortality rates by as much as 46% [12]. Despite this, real-world data demonstrate underutilization of NIV in AECOPD. National audits from the UK continue to report variation in NIV utilization among eligible patients, while large US cohorts demonstrate increasing NIV adoption over time and considerable hospital-level variation in clinical practice [8–10, 13, 14]. In our cohort, NIV was widely accessible, with 80% of patients receiving it at some point during their ICU stay; however, its overall contribution remained limited, accounting for only 25% of total ventilation time.

This short overall NIV duration recorded in our cohort is further explained by the NIV failure rate (53.3%), which is notably high compared to both international literature and a previously published report from an expert center in the region [11, 20]. Only baseline heart rate was associated with need for conversion in our cohort, and PaCO_2_ was not a predictor of NIV failure, despite extensive literature on the subject. This is more likely a result of the small sample size and severe morbidity of the cohort. Previous studies have suggested that change in pH and PaCO_2_ as a result of NIV might be a better predictor of therapeutic success, which is directly influenced by the adequacy of ventilator settings [7]. As serial blood gas measurement and ventilator settings were not collected in our study, this hypothesis could not be evaluated in our cohort. However, the predominance of early NIV failure in particular might point to inadequate patient selection or suboptimal ventilation settings, rather than timing or patient severity alone [20]. GCS was the strongest determinant of both non-survival status and initial IMV, while PaCO_2_ or pH had no prognostic effect in these comparisons, suggesting that impaired consciousness caused by other causes (possibly sepsis and multi-organ involvement) is a predictor of poor outcome and a strong clinical signal to avoid NIV. The high occurrence of early NIV failure in non-survivors in our cohort may indicate inappropriate patient selection and delayed intubation in this patient group.

Although improperly applied NIV settings have been proven to increase morbidity and mortality, there is a lack of clear clinical practice guidelines, particularly regarding therapy goals and adjustment of ventilator parameters [28]. Limited clinical experience may also play a role, although the more successful application of NIV as a step-down therapy in our cohort suggests growing familiarity and confidence with this therapy.

Strengths of this study include its prospective design, multicenter data collection, and external data contextualization. It provides real-world insights regarding treatment patterns and outcomes of the most severe AECOPD cases in the region, however, several limitations need to be recognized. First, this was an exploratory study conducted during a predefined two-month enrollment period and no a priori sample size calculation was performed. Consequently, the overall sample size was small, increasing the risk of both type I and type II statistical errors and limiting the interpretation of subgroup analyses, including sex-related differences. Second, although the multicenter design reflects routine clinical practice, no study-specific protocol was implemented regarding NIV practice. Clinical decisions were made according to local practice, which may have introduced inter-center variability and affected outcomes. Third, a small proportion of patients were included based on probable, rather than documented COPD diagnosis, which may have contributed to population heterogeneity. Fourth, no detailed data was collected regarding clinical changes during NIV therapy, or weaning practice, limiting the generalizability of these results. Fifth, a small number of patients may have been managed in emergency departments or others wards, and were therefore not enrolled, as the study included only patients admitted to the participating ICUs. Sixth, reasons for NIV failure were not systematically collected, limiting to explore the mechanisms behind NIV failure. Seventh, no standardized protocols were used during the study, which may have introduced inter-center and inter-clinician variability.

Finally, the interpretation of the NHIF comparison should be regarded with caution, as administrative claims data are subject to coding inaccuracies and the nationwide hospitalized COPD population is not directly comparable to the ICU cohort. Nevertheless, the multicenter prospective design provides important real-world data and highlights several areas for future investigation and quality improvement. Larger studies with standardized data collection are needed to better evaluate current practice and their impact on outcome.

## Conclusion

Severe AECOPD in the Hungarian ICU setting is currently associated with high NIV failure rate and considerable hospital mortality. The lack of predictive markers for NIV failure and the high early failure rate in particular underscores the need for more nuanced clinical guidelines, including ventilator optimization and monitoring of patient trajectories. Future studies should focus on appropriate patient selection, initial NIV application and therapy optimization.

## Supplementary Information

Supplementary Material 1: Supplementary Table 1: Comparison of initial ventilation modality. Table legend: Values are presented as median [interquartile range] or number (%), as appropriate. Bold values indicate statistical significance (*p* < 0.05). (BMI: body mass index, PaO₂: arterial partial pressure of oxygen, PaCO₂: arterial partial pressure of carbon dioxide,HCO₃⁻: bicarbonate, FiO₂: fraction of inspired oxygen, GCS: Glasgow Coma Scale, APACHE II: Acute Physiology and Chronic Health Evaluation II, NIV: noninvasive ventilation, IMV: invasive mechanical ventilation, ICU: intensive care unit, N: number).

## Data Availability

The datasets used and analyzed during the current study are available from the corresponding author on reasonable request.

## Abbreviations

AECOPD: Acute exacerbation of chronic obstructive pulmonary disease
APACHE II: Acute Physiology and Chronic Health Evaluation II
BMI: Body mass index
COPD: Chronic obstructive pulmonary disease
FiO₂: Fraction of inspired oxygen
GCS: Glasgow Coma Scale
HCO₃⁻: Bicarbonate
ICU: Intensive care unit
IMV: Invasive mechanical ventilation
MAP: Mean arterial pressure
NHIF: National Health Insurance Fund
NIV: Noninvasive ventilation
N: Number
PaCO₂: Arterial partial pressure of carbon dioxide
PaO₂: Arterial partial pressure of oxygen
