# Supplementary material for: Real-world treatment patterns and outcomes in severe acute exacerbations of chronic obstructive pulmonary disease: an observational study
Source: BMC Pulm Med. 2026 Aug 14;26:552. doi: 10.1186/s12890-026-04599-z (PMC13753241; doi:10.1186/s12890-026-04599-z)
Supplement: Supplementary file 1 [file 12890_2026_4599_MOESM1_ESM.docx]

Supplementary Table 1: Comparison of initial ventilation modality

|  | **NIV**  N=15 | **IMV**  N=12 | p-value |
| --- | --- | --- | --- |
| Age (years) | 66.5 (55-73) | 70 (66-76) | 0.581 |
| BMI (kg/m^2^) | 28.3 (24.7-36.7) | 29.4 (24.2-32.7) | 0.719 |
| Systolic blood pressure (mmHg) | 128 (107-150) | 90 (82-107) | 0.083 |
| Diastolic blood pressure (mmHg) | 71 (61-74) | 49 (42-56) | 0.083 |
| Mean arterial pressure (mmHg) | 91 (74.3-102) | 62.3 (55-69.3) | **0.047** |
| Heart rate (/min) | 86.5 (70-105) | 98 (82-110) | 0.829 |
| pH | 7.22 (7.15-7.29) | 7.29 (7.27-7.31) | 0.139 |
| PaO_2_ (mmHg) | 68.4 (59.8-147) | 75.8 (64.2-82) | 0.373 |
| PaCO_2_ (mmHg) | 89.3 (76.5-109) | 58.7 (51.9-71.6) | **0.05** |
| HCO_3_^-^ (mmol/L) | 36.9 (29.3-39) | 29.3 (27.5-33.5) | **0.002** |
| Respiratory rate (/min) | 25.5 (15-32) | 23 (18-26) | 0.867 |
| Body temperature (°C) | 36.5 (36-36.9) | 36.3 (36-37) | 0.399 |
| FiO_2_ (%) | 50 (40-75) | 80 (60-100) | 0.323 |
| GCS (3-15) | 14 (13-15) | 3 (3-6) | **0.04** |
| Serum sodium (mmol/L) | 139.5 (132-142) | 138 (136-140) | 0.981 |
| Serum potassium (mmol/L) | 4.6 (3.9-5.3) | 4.6 (4-4.8) | 0.373 |
| Serum creatinine (mmol/L) | 96.5 (87-141) | 107 (93-130) | 0.981 |
| Hematocrit (%) | 41 (36-51) | 42 (38-44) | 1.000 |
| White Blood Cell Count (x10^3^ /µL) | 12.8 (8.9-14.2) | 11 (9.8-15.2) | 0.867 |
| APACHE II Points | 19.5 (17-26) | 27 (26-32) | 0.126 |
| APACHE II Estimated Nonoperative Mortality (%) | 32.5 (25-55) | 55 (55-73) | 0.083 |
| Duration of NIV (days) | 1 (1-2) | 1 (0-2) | 0.126 |
| Duration of IMV (days) | 0.5 (0-5) | 5 (3-14) | **0.037** |
| ICU length of stay (days) | 5.5 (3-8) | 6 (4-14) | 0.256 |
| In-hospital survival | 11 (73.3%) | 6 (50%) | 0.212 |
| ICU survival | 12 (80%) | 9 (75%) | 0.756 |

Table legend: Values are presented as median [interquartile range] or number (%), as appropriate.

(BMI: body mass index, PaO₂: arterial partial pressure of oxygen, PaCO₂: arterial partial pressure of carbon dioxide,HCO₃⁻: bicarbonate, FiO₂: fraction of inspired oxygen, GCS: Glasgow Coma Scale, APACHE II: Acute Physiology and Chronic Health Evaluation II, NIV: noninvasive ventilation, IMV: invasive mechanical ventilation, ICU: intensive care unit, N: number)
